# Root iron uptake efficiency of* Ulmus laevis* and *U. minor* and their distribution in soils of the Iberian Peninsula

**DOI:** 10.3389/fpls.2014.00104

**Published:** 2014-03-25

**Authors:** Martin Venturas, Victoria Fernández, Paloma Nadal, Paula Guzmán, Juan J. Lucena, Luis Gil

**Affiliations:** ^1^Forest Genetics and Ecophysiology Research Group, School of Forest Engineering, Technical University of MadridMadrid, Spain; ^2^Departamento de Química Agrícola, Facultad de Ciencias, Universidad Autónoma de MadridMadrid, Spain

**Keywords:** calcifuge, iron chlorosis, iron reductase activity, nutrition, root proton extrusion, *Ulmus*

## Abstract

The calcifuge and calcicole character of wild plants has been related to nutrient availability shortages, including iron (Fe)-deficiency. Surprisingly, just a few studies examined the relation between root Fe uptake and plant distribution in different soil types. We assessed the root Fe acquisition efficiency of two *Ulmus* species with calcareous (*Ulmus minor)* and siliceous (*U. laevis*) soil distribution patterns in the Iberian Peninsula. Seedlings of both elm species were grown hydroponically with different Fe concentrations during 6 weeks. Plant physiological responses to Fe-limiting conditions were evaluated as were the ferric reductase activity and proton (H^+^) extrusion capacity of the roots. Iron deprived elm seedlings of both species were stunted and suffered severe Fe-chlorosis symptoms. After Fe re-supply leaf chlorophyll concentrations rose according to species-dependent patterns. While *U. minor* leaves and seedlings re-greened evenly, *U. laevis* did so along the nerves of new growing leaves. *U. minor* had a higher root ferric reductase activity and H^+^-extrusion capability than *U. laevis *and maintained a better nutrient balance when grown under Fe-limiting conditions. The two elm species were found to have different Fe acquisition efficiencies which may be related to their natural distribution in calcareous and siliceous soils of the Iberian Peninsula.

## INTRODUCTION

**FIGURE 1:**
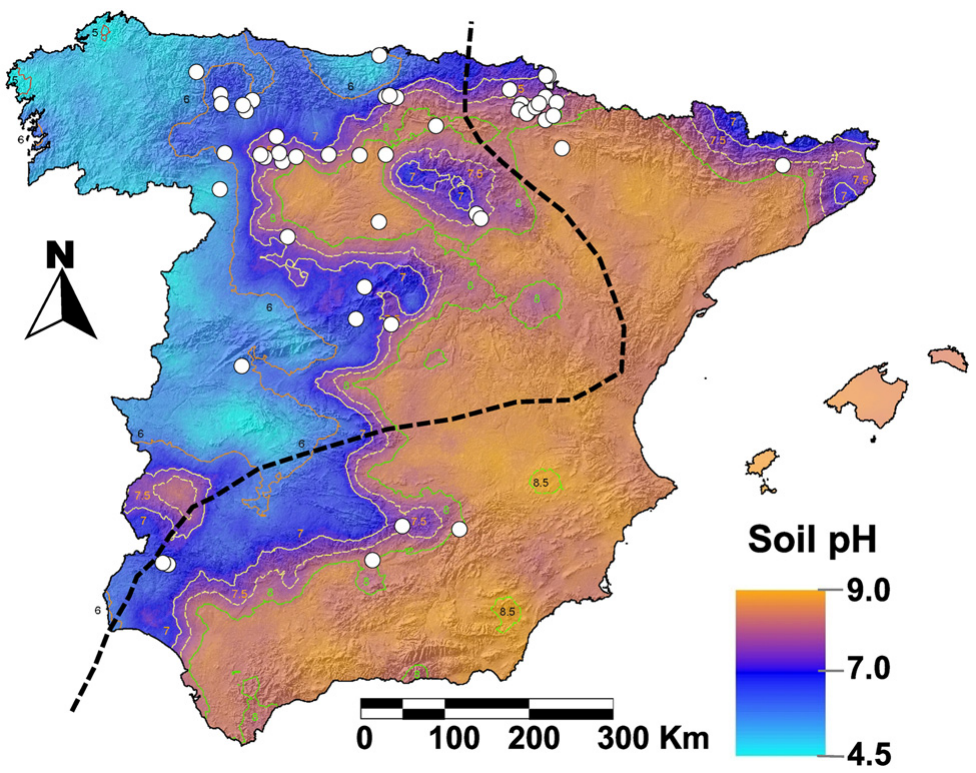
***Ulmus laevis *populations (Venturas et al., 2013a) represented as white dots over an Iberian Peninsula soil pH map (Rodríguez et al., 2009).** *Ulmus minor* is thought to be native east of the broken line (Richens and Jeffers, 1986), although it grows all over Spain because this species has been extensively used by humans (Gil et al., 2004).

*Ulmus laevis* is a Northern Hemisphere genus of importance in the ecological context of the Iberian Peninsula (García-Nieto et al., 2000). The indigenous elm species found in Spain are *U. glabra *Huds, *U. laevis *Pall. and *U. minor *Mill. The native character of *U. laevis* has been recently confirmed by DNA molecular markers (Fuentes-Utrilla et al., 2014). Despite *U. minor* being native to Spain (Richens and Jeffers, 1986; Gil and García-Nieto, 1990), its natural distribution is not clear since this species has been extensively planted for over 2,000 years, first for training vines and later for ornamental purposes (Gil et al., 2004). *U. minor* can be found in the whole Iberian Peninsula in azonal flood-plain forests, linked to shallow water-tables, since it tolerates floods as well as summer droughts. Nonetheless, it preferentially grows in Eastern Spain (**Figure 1**) were there is an abundance of calcareous, alkaline soils (Richens and Jeffers, 1986). In contrast, *U. laevis* is one of the few European tree species which thrives in damp soils that are seasonally flooded (Collin et al., 2000). In Spain its relic populations are scarce, small and fragmented (**Figure 1**, Venturas et al., 2013a; Fuentes-Utrilla et al., 2014). *U. laevis* chiefly grows in acid soils of the Western Iberian Peninsula as riparian forests, which are subjected to seasonal waterlogging and linked to aquifer discharge areas and/or endorheic basins.

The distribution of *U. laevis* and *U. minor* in the Iberian Peninsula may be related to calcifuge versus calcicole characteristics of each species. Whilst calcicole plants are able to take up nutrients when grown in calcareous soils (Zohlen, 2002), calcifuge species growth under high pH, alkaline soil conditions is limited by low nutrient availability especially phosphorus (P; Tyler, 1992; Zohlen and Tyler, 2004), iron (Fe; Zohlen and Tyler, 1997, 2000; Zohlen, 2002), and manganese (Mn; Messenger, 1986; Thomas et al., 1998; Kuster et al., 2013).

Iron is a vital element for plant growth, development and survival, since it is essential for the proper functioning of multiple metabolic and physiological processes (López-Millán et al., 2013; Sudre et al., 2013; Grillet et al., 2014). The detrimental effect of lime-induced chlorosis on wild plants grown in calcareous, high pH soils has been only analyzed in a few eco-physiological studies (Hutchinson, 1970; Anderson, 1984; Tyler, 1996; Zohlen and Tyler, 1997; Zohlen, 2002). While different plant strategies and specific mechanisms for the acquisition and homeostasis of Fe have been characterized for several agronomic crops (Römheld, 1987; Tagliavini and Rombolà, 2001; Kobayashi and Nishizawa, 2012; Roschzttardtz et al., 2013), there is limited information on the performance of forest species (Gogorcena et al., 2001; Fodor et al., 2005; Pestana et al., 2012; Kuster et al., 2013).

Despite the ubiquitous presence of Fe in the earth’s crust, the low solubility of Fe(III) compounds in many soils prevents plant Fe uptake and induces the development of Fe deficiency symptoms (Lindsay, 1984; Lucena, 2006). Iron chlorosis is principally observed in calcifuge plants growing in calcareous soils, where CaCO_3_ buffers soil solution pH in the range of 7.5–8.5 (Lindsay and Schwab, 1982), and in the presence of high bicarbonate concentrations (Lucena, 2000). According to the specific root uptake mechanisms for the acquisition of Fe, plants have been classified as (Marschner and Römheld, 1994): Strategy I (dicotyledoneous and non-graminaceous monocotyledoneous species) or Strategy II (restricted to graminaceous species), which are additionally capable of producing phyto-siderophores. Iron uptake mechanisms in Strategy I plants may involve metabolic and morphological changes (Rombolà et al., 2002; Abadía et al., 2011; López-Millán et al., 2013), but the major components are: (i) the occurrence of a Fe-reductase enzyme of the FRO (membrane-bound ferric reductase) family, (ii) the induction of an IRT (iron regulated transporter) Fe(II) transporter that belongs to the ZIP family (ZRT, IRT-like protein), (iii) acidification of the rhizosphere via the excretion protons by a H^+^-ATPase, and (iv) the excretion of organic compounds, such as carboxylates, phenolics, and flavonoids, which can affect Fe-availability directly or indirectly. The relative importance and efficacy of the different root Fe uptake mechanisms may vary among plant species, varieties and populations, which may be associated with the tolerance or susceptibility when grown in high pH, calcareous soils (Dell’Orto et al., 2013).

Information on the root response mechanisms of trees to lime-induced chlorosis is mostly limited to some fruit species such as peach (Gogorcena et al., 2000; Jiménez et al., 2011), *Annona* (Ojeda et al., 2003), olive, pear, quince (De la Guardia and Alcántara, 2002; Donnini et al., 2011), and to a few forest or woody species like cork-oak (Gogorcena et al., 2001), poplar (Fodor et al., 2005), carob and trifoliate orange (Pestana et al., 2012). On the other hand, the response of some calcicole and calcifuge plants to lime-induced chlorosis (e.g., Zohlen and Tyler, 1997, 2000; Zohlen, 2002; Donnini et al., 2012) has been principally evaluated on the basis of tissue Fe determinations.

Therefore, given the markedly different distribution of *U. laevis* and *U. minor* in siliceous and calcareous soils of Spain and as a first approach, a greenhouse study was undertaken to assess the effect of growing these species under hydroponic, Fe-limiting conditions. With the aim of comparing the performance of both elm species under Fe-limiting conditions, we tested the following hypotheses: (i) Do *U. laevis* and *U. minor* differ in their degree of tolerance to lime-induced chlorosis in relation to growth, deficiency symptom development and nutrient homeostasis?, and (ii) is *U. minor* more efficient than *U. laevis* in acquiring Fe via the root system as measured by ferric reductase activities and proton extrusion capacities?

## MATERIALS AND METHODS

### PLANT CULTURE

*Ulmus minor *P-VV1 genotype seeds (the code refers to the Spanish Elm Breeding Program) were collected at Puerta de Hierro nursery (Madrid, 40°27’N, 3°45’W) conservation plot, and *U. laevis *M-VD.R29 genotype seeds (referring to the Spanish Elm Breeding Program) at Valdelatas forest (Madrid, 40°32’N, 3°40’W). In the first experimental week, seeds were germinated in 4 L pots filled with perlite. All the materials used for plant growth (perlite and pots) were previously washed (two washes in 0.1 M HCl, followed by a wash in 100 μM ethylenediaminetetraacetic acid disodium (Na_2_EDTA) and several rinses in pure water) to limit the potential occurrence of Fe contamination. Seeds were initially irrigated with distilled water (type II analytical grade, obtained with an Elix 5 apparatus, Millipore, USA) until the development of the first true leaf (c.a. 3 weeks after seed sowing). Seedlings were subsequently grown in ¼ strength nutrient solution (T0, as described below) for 1 week, ½ strength for another 2 weeks, and finally in full-strength solution until the end of the experimental period. The base nutrient solution without Fe (T0) was changed once per week and had a composition of: (i) macronutrients (mM): 1.0 Ca(NO_3_)_2_, 0.9 KNO_3_, 0.3 MgSO_4_, and 0.1 KH_2_PO_4_; (ii) EDTA-buffered metal micronutrients (μM): 2.5 MnSO_4_, 1.0 CuSO_4_, 10.0 ZnSO_4_, 1.0 NiCl_2_, 1.0 CoSO_4_, and 115.5 Na_2_EDTA; and (iii) other micronutrients (μM): 35 NaCl, 10 H_3_BO_3_, and 0.05 Na_2_MoO_4_ (Nadal et al., 2012). The pH of the solution was buffered with 4-(2-hydroxyethyl)-1-piperazineethanesulfonic acid (HEPES) at 0.1 mM and adjusted to 7.5 with 1.0 M KOH (Nadal et al., 2012). Seedlings were grown in a greenhouse under a 16/8 h day/night regime and temperatures ranging from 10 to 25°C. A total of 72 homogeneous seedlings from the two species were selected and transferred to hydroponic culture 1 month after sowing. Twelve plants from the same species were placed together in continuously aerated (approximately 100 L of air per hour and container) 6 L containers filled with full-strength nutrient solution.

### TREATMENT APPLICATION

When seedlings were 2 months old the nutrient solution was changed for the onset of the different root Fe treatments. When supplied to the plants, iron was applied as Fe(III)-HBED previously synthesized in the laboratory by complexing free Fe(III) supplied as Fe(NO_3_)_3_ 9H_2_O with HBED (ADOB PPC, Poznan, Poland) at 1:1 (Fe:ligand) ratios.

Three different Fe treatments per species were initially added to the base nutrient solution, namely: 0 μM Fe (i.e., T0 containing no Fe, as described above), 5 μM Fe (T5) and 20 μM Fe (T20). Four containers per species received 0 μM Fe (T0). Nutrient solutions were changed once per week until the end of the experimental period (after 6 weeks). To achieve a Fe-deficiency state similar to what may occur to Fe-chlorotic field trees and an intermediate chlorosis level, an additional 1 μM Fe treatment (T1) was given for 2 weeks to one T0 container per species (i.e., to 12 plants).

### PLANT PHYSIOLOGICAL RESPONSES

After Fe-resupply, plant heights and SPAD values (measured with a SPAD 502, Minolta, Osaka, Japan) were determined on a weekly basis for 6 weeks on 12 plants per treatment and species. For SPAD index determinations, which give an estimation of leaf chlorophyll (Chl) concentrations (Nadal et al., 2012), two sub-apical leaves per plant were measured, with two measurements per leaf. Total Chl contents were determined by the method of McKinney (1941) and correlations with SPAD values were established for both species. In brief, Chl concentrations were measured from fresh tissue after extraction in 80% acetone and spectrophotometric reading at 664 nm for Chl a and 647 nm for Chl b (7315 Spectrophotometer Jenway, Bibby Scientific Limited, UK).

Plant tissues were analyzed following standard laboratory procedures and limiting the risk of Fe contamination by rising the flasks, test tubes, filter paper, funnels and caps in 0.1 M HCl prior to use (Nadal et al., 2012). At the end of the experimental period, all the leaves and stems of the plants were separated and thoroughly washed with 0.1% HCl plus 0.05% surfactant (Tween 20, Sigma Aldrich). They were then rinsed once in tap water and twice in distilled water (Nadal et al., 2012). The mineral element composition of *Ulmus* seeds was also analyzed after separation of the seeds from the samaras. Plant tissues were subsequently oven-dried at 70°C for 2 days, weighed and ground prior to mineral element determination after dry-ashing. Carbon and N were measured with an elemental analyser (TruSpec, Leco Corporation, St. Joseph, MI, USA). The remaining elements were determined by inductively coupled plasma (ICP, Perkin-Elmer, Optima 3000) following the UNE-EN ISO/IEC 17025 standards for calibration and testing laboratories (CEBAS-CSIC Analysis Service, Murcia, Spain).

### ROOT PROTON EXCRETION

To assess the acidification (proton extrusion; Römheld et al., 1984) capacity of the roots of *U. minor* and *U. laevis*, 15 plants (10 weeks old) per species never supplied with Fe via the root system (i.e., belonging to the T0 treatment) were selected. Individual plants were transferred to 250 mL dark, sterile, aerated plastic jars. Each black lid closing a jar had two different size perforations to allow insertion of an air dispersion tube, and an elm seedling. Three treatments without HEPES were applied (five replicates per Fe-treatment and species): T0, T5, and T20 solutions and adjusting the pH to 7.0 with 1.0 M KOH. Each jar was filled with 200 mL of its corresponding nutrient solution. At 1, 2, 3, 4, 7, 8, and 9 days after the beginning of the treatment (DAT) the jars were first filled up to 200 mL with pure water, and a 5 mL sample was collected for titration. Twenty-five mL of pure water were added to each 5 mL nutrient solution sample. Root H^+^ extrusion was subsequently calculated after titration of the diluted nutrient solution with 0.5 mM NaOH to reach again the initial pH of 7.0 (measurements carried out with a Micro-pH 2002 pH-meter, Crison Instruments, Spain). Since solutions were continuously aerated, the contribution of root respiration to solution acidification was negligible. Shoot and root fresh weights (FWs) were recorded at the end of the experimental period. The daily H^+^ extrusion per plant (μmol *g*^-1^ root FW day^-1^) was calculated as the H^+^ increment since the previous day.

### ROOT REDUCTASE ACTIVITY

The method described by Lucena and Chaney (2006) was followed to assess the root ferric reductase activity (RA) of the plants. Two days before RA measurement, the 6 L containers were transferred to a growth chamber (with 30°C/25°C day/night temperatures) with cool-white fluorescent and incandescent light (200 μEm^-2^ at plant height during 16 h per day). Two-hundred-and-fifty mL darkened, sterile plastic jars with black lids were placed in the growth chamber. Each jar contained 200 mL reductase assay solution consisting of: macronutrient solution T0 as in the growth period; 100 μM Fe(III)-EDTA used as substrate of the ferric chelate reductase; 2 mM 2-morpholinoethanesulfonic acid (MES) to buffer the pH at 6.0; and 300 μM bathophenanthrolinedisulfonic acid (BPDS) as an Fe^2+^ trapping and coloring reagent. The lids had one hole for a plastic gas dispersion tube, another for a pipette, and a third one to hold one elm seedling. Each solution was continuously aerated and allowed to reach temperature equilibrium before plants were transferred. Experiments began 3 h into the daylight period. Initial 3 mL samples were obtained for each jar. The roots of plants were washed three times in macronutrient solution containing 37.5 μM Na_2_BPDS, and then transferred to the RA solutions. Seven replicates (14 weeks old plants) were prepared for each treatment (T0, T1, and T20) and species. Four jars without plants were included in order to correct reduction rates for slow photo-reduction. Three-mL nutrient solution samples were withdrawn at 10, 20, 60, and 120 min and the Fe(II)-BPDS concentration was subsequently measured at 535 nm, with a V-650 spectrophotometer (Jasco, Easton, MD, USA). Standard Fe(II)-BPDS solutions were previously prepared and molar absorption coefficients at 535 nm were determined. For each experimental plant, the ferric RA activity was determined as the slope of the curve resulting from plotting the Fe(II) concentration recorded over time divided by the root FW.

### STATISTICAL ANALYSIS

Analyses of variance (ANOVA) were performed for SPAD values, plant height, root proton extrusion, reductase activity, Chl and tissue mineral element concentrations. The factors considered were Fe-treatments nested within species. All pairwise comparisons of mean values were performed using Tukey’s honestly-significant difference (HSD) multiple range test at a 95% confidence level. All statistical analyses were performed using STATISTICA 7.0 software (StatSoft Inc., Tulsa, OK, USA).

## RESULTS

### PLANT PHYSIOLOGICAL RESPONSES

**FIGURE 2:**
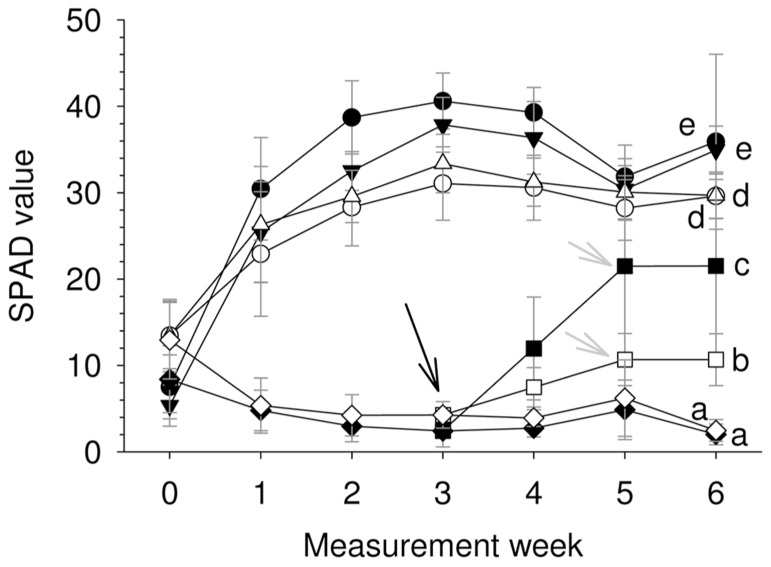
**SPAD evolution for the different treatments during 6 weeks after Fe-resupply to 2 months-old plants (*n* = 12, mean ± SD; letters correspond to Tukey’s HSD 95% homogeneous groups).** Filled symbols, *U. laevis*; empty symbols, *U. minor*; circles, 20 μM Fe; triangles, 5 μM Fe; squares, 1 μM Fe; diamonds, 0 μM Fe. The black arrow indicates when the 1 μM treatment was initiated and gray arrows when it was stopped.

For the two species, Fe-supply led to a steep Chl concentration increment during the first 2 weeks (**Figure 2**), which was maintained until the end of the experimental period. In general and for a similar root Fe supply level *U. laevis* had higher SPAD values than *U. minor* leaves, and there were no significant differences between the 20 and 5 μM Fe treatments at the end of the trial. The supply of 1 μM Fe to Fe-deficient seedlings (indicated with an arrow) in week 3 led to a steep Chl increase (**Figure 2**).

**FIGURE 3:**
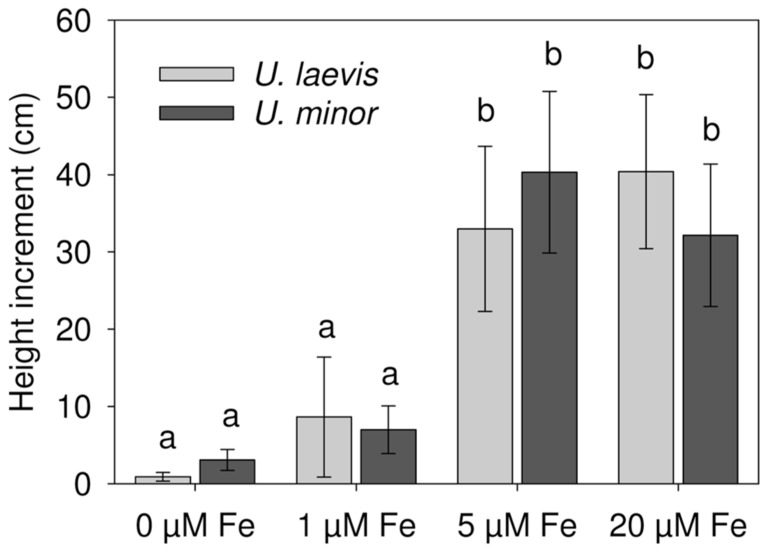
**Plant height increment (Week 6 – Week 0) after Fe-resupply to 2 months-old plants (*n* = 12, mean ± SD, letters correspond to Tukey’s HSD 95% homogeneous groups)**.

Both elm species fully supplied with iron (20 μM Fe treatment) were green, had a healthy appearance and a large size (especially *U. laevis*) during the whole experimental period (**Table 1**, **Figure 3**). For this treatment, *U. laevis* leaves had 37% more total Chl concentrations than *U. minor* (**Table 1**). Iron-sufficient, *U. laevis* leaves also had 34% higher tissue Fe concentrations than *U. minor* leaves (**Table 1**).

**Table 1:** SPAD values, leaf chlorophyll (Chl) and iron leaf and stem concentrations of *U. laevis *and *U. minor* seedlings 6 weeks after Fe-resupply.

Species	Treatment	Mean aerial part DW (*g*)	Leaf			Stem
			Δ SPAD	[Fe] (mg Kg^-1^)	Total [Chl](mg cm^-2^)	[Fe] (mg Kg^-1^)
***U. laevis***	0 μM Fe	0.10 ± 0.02 a	^-^6.4 ab	21.0 ± 0.4 a	0.08	6.2 ± 0.1 b
	1 μM Fe	0.47 ± 0.13 b	14.4 c	23.8 ± 5.8 ab	1.98	8.1 ± 0.2 bc
	5 μM Fe	1.95 ± 0.30 c	29.6 d	37.0 ± 3.6 bc	2.73	10.8 ± 1.3 c
	20 μM Fe	2.07 ± 0.51 c	28.4 d	60.3 ± 2.7 d	3.01	13.7 ± 1.0 cd
***U. minor***	0 μM Fe	0.23 ± 0.15 a	^-^10.5 a	10.8 ± 0.2 a	0.13	4.3 ± 0.1 a
	1 μM Fe	0.34 ± 0.18 a,b	^-^2.6 b	11.9 ± 0.1 a	0.70	9.0 ± 0.2 c
	5 μM Fe	2.81 ± 1.57 c,d	16.3 c	38.8 ± 2.3 bc	2.12	9.9 ± 0.8 c
	20 μM Fe	1.23 ± 0.35 c	16.2 c	44.9 ± 1.5 c	2.19	15.0 ± 0.9 d

*The headings correspond to: mean aerial part dry weight (DW; *g*), Δ SPAD (SPAD increment of Week 6 – Week 0; *n* = 12), total leaf [Chl], and leaf and stem [Fe] (mg kg^-1^DW; means ± SE; *n* = 7). Letters correspond to Tukey’s HSD 95% homogeneous groups.*

**FIGURE 4:**
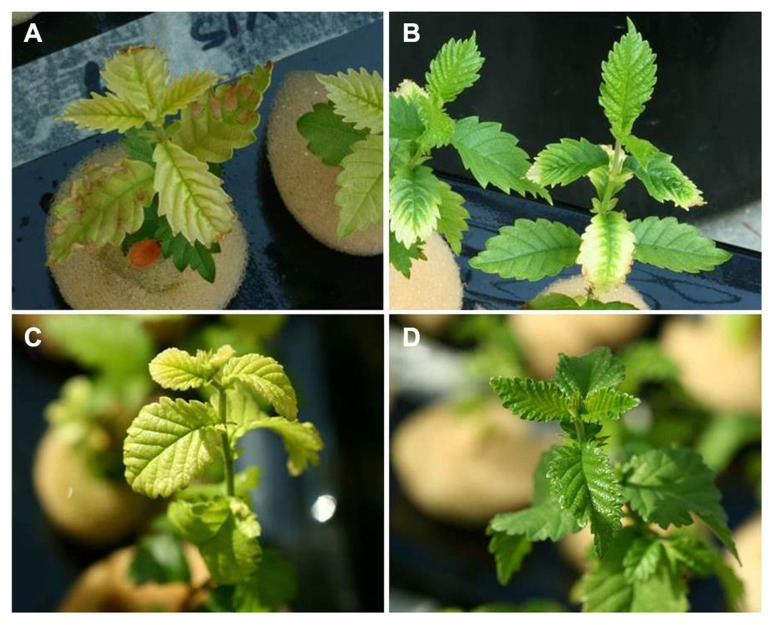
**Leaf appearance of Fe chlorotic and Fe-resupplied 3 months-old elm seedlings.** *Ulmus laevis: ***(A)** chlorotic leaves, **(B)** re-greening pattern after Fe supply. *Ulmus minor*: **(C)** chlorotic leaves, **(D)** re-greening pattern after Fe-supply.

There were significant differences in SPAD values between the species and Fe treatments after 6 weeks (*P *< 0.0001; **Table 1**, **Figure 2**). Both elm species developed strong Fe-chlorosis symptoms in the lack of Fe. The seedlings subjected to the 0 μM Fe treatment were stunted and leaves were severely chlorotic with necrotic spots, chiefly in the case of *U. laevis *(**Figures 3** and **4A,C**).Once supplied Fe, the re-greening patterns of seedlings and leaves were different in both species. The re-greening process in *U. laevis *leaves began along the veins of apical leaves and basal leaves took longer to re-green and never reached a homogeneous green coloration (**Figure 4B**). Meanwhile, *U. minor* re-greened evenly all over the plant and leaf surface (**Figure 4D**). Seedlings of the 5 and 20 μM Fe treatments reached their maximum SPAD values in week 3 (**Figure 2**). Regarding tree height and for both species, no significant differences were found between the 5 and 20 μM Fe treatments, while plants receiving 1 and 0 μM Fe in the nutrient solution had a stunted growth (**Figure 3**).

### ROOT PROTON EXCRETION

The proton extrusion per day was significantly different between the species (*P *< 0.0001), but was not found to be significant for treatments nested within species (*P *= 0.57). The highest rate of proton excretion was measured on the first day of the trial when 280 and 97 μmol H^+^ *g*^-1^ day^-1^ were recorded for *U. minor* and *U. laevis*, respectively. During the following 8 days, root extrusion values decreased to reach a steady average of 17 (*U. minor*) and 9 (*U. laevis*) μmol H^+^ *g*^-1^ day^-1^.

### ROOT FERRIC REDUCTASE ACTIVITY

**FIGURE 5:**
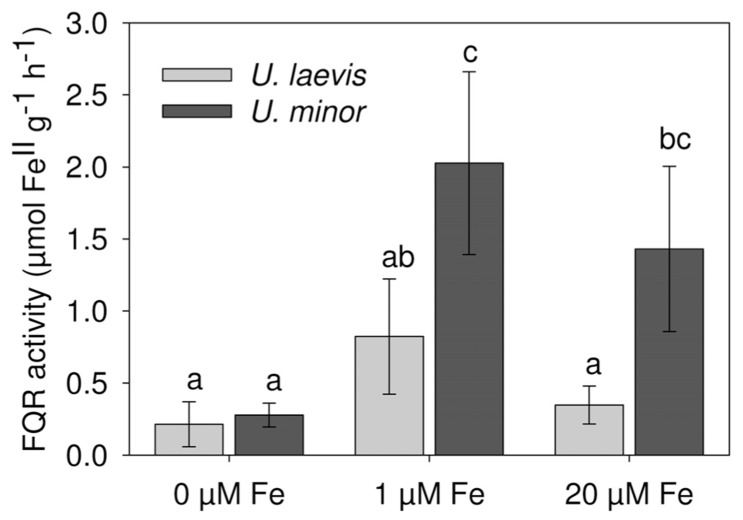
**Root ferric reductase activity (FQR) of 3 months-old *U. laevis* and *U. minor* plants (*n* = 7, means ± SD, letters correspond to Tukey’s HSD 95% homogeneous groups)**.

The root ferric reductase activity was significantly different between species (*P *= 0.0004) and treatments nested within species (*P *< 0.0001; **Figure 5**). For the 1 μM Fe and 20 μM Fe treatments, *U. minor* had a much higher reductase activity than *U. laevis*. However during 2 h of the assay, Fe-deficient (0 μM Fe) plants of both species had a limited root ferric reductase activation capacity in the presence of Fe(III) in the nutrient solution (**Figure 5**).

### MINERAL ELEMENT STATUS

The macro- and micro-nutrient concentrations of leaves and stems in relation to the different treatments are shown in **Tables 1–3**. Leaf and stem Fe concentrations decreased under Fe-limiting conditions (**Table 1**). At the end of the experimental period and as derived from **Figure 3**, the aerial part dry weight (DW) gradually increased with higher root Fe levels for *U. laevis*. While a similar trend was observed for *U. minor* seedlings, the highest mean aerial plant DW was recorded for the 5 μM Fe treatment, which however, had a high SD (**Table 1**). This implies that the highest Fe absorption rates were achieved by the 20 μM Fe *U. laevis *and 5 μM Fe *U. minor* treated seedlings, suggesting that the later species possibly reached an optimum when supplied 5 μM Fe via the root system.

For all elements excepting K, Fe, and Zn in leaves and K and Fe in stems, variations in *U. laevis *were larger than in *U. minor*. For both species, the relative changes followed a similar trend for all the elements excepting Zn, which increased in *U. laevis *chlorotic plants and decreased in *U. minor *(**Tables 2** and **3**). Leaf N concentrations decreased with lower root Fe supply levels, especially in *U. laevis*. On the contrary, stem N concentrations were higher in seedlings of the 0 μM Fe treatment. Phosphorus tissue concentrations increased in association with lower root Fe doses. Potassium leaf and stem concentrations were generally lower in 0 μM Fe treated elm seedlings, which also had the lowest leaf Ca concentrations (**Table 2**). Concerning the micronutrients, approximately five and eight times higher Mn concentrations were determined in leaves and stems of Fe-deprived versus 20 μM Fe treated *U. laevis* seedlings, indicating the accumulation of Mn under Fe-limiting conditions. However, in *U. minor* the lack of Fe in the growing medium approximately doubled the Mn concentration of leaves and stems as compared to the 20 μM Fe treatment. Root Fe shortage also led to the accumulation of B in leaves and of Cu in leaves and stems while no clear trend was observed for Zn concentrations in leaves and stems in relation to the different Fe treatments (**Table 3**).

**Table 2:** Macronutrient leaf and stem concentrations (mean ± SE; the same letters within the same tissue and element correspond to Tukey’s HSD 95% homogeneous groups).

			[Macronutrients] (*g* kg^-1^ DW)				
Tissue	Species	Treatment	N	P	K	Ca	Mg
**Leaf**	***U. laevis***	0 μM Fe	25.8 ± 0.5 a	9.3 ± 0.2 f	16.8 ± 0.3 ab	53.0 ± 1.1 d	10.4 ± 0.2 e
		1 μM Fe	30.7 ± 0.3 bc	6.0 ± 0.2 e	17.5 ± 1.1 ab	38.4 ± 1.7 cd	6.8 ± 0.2 d
		5 μM Fe	32.1 ± 3.4 bc	2.6 ± 0.1 ab	14.0 ± 0.7 a	32.2 ± 2.0 abc	5.0 ± 0.3 c
		20 μM Fe	40.1 ± 0.4 c	2.4 ± 0.1 ab	15.9 ± 0.7 ab	25.2 ± 1.2 a	3.5 ± 0.2 a
	***U. minor***	0 μM Fe	35.3 ± 0.7 bc	5.2 ± 0.1 de	15.0 ± 0.3 a	40.0 ± 0.8 cd	5.6 ± 0.1 cd
		1 μM Fe	34.3 ± 1.6 bc	4.1 ± 0.5 cd	17.2 ± 1.5 ab	36.8 ± 1.0 bc	6.5 ± 0.1 d
		5 μM Fe	37.4 ± 1.0 bc	2.4 ± 0.1 a	15.9 ± 1.2 ab	32.6 ± 1.9 bc	4.6 ± 0.2 bc
		20 μM Fe	39.7 ± 0.9 c	3.2 ± 0.2 bc	19.3 ± 1.1 b	27.9 ± 1.5 ab	3.6 ± 0.3 ab
**Stem**	***U. laevis***	0 μM Fe	32.5 ± 0.6 b	4.7 ± 0.1 d	9.6 ± 0.2 a	20.1 ± 0.4 c	2.0 ± 0.0 bc
		1 μM Fe	18.0 ± 0.4 a	3.6 ± 0.1 cd	12.4 ± 0.2 ab	14.1 ± 0.3 bc	1.3 ± 0.0 abc
		5 μM Fe	19.5 ± 2.8 a	1.6 ± 0.1 ab	12.5 ± 0.9 ab	8.9 ± 0.7 ab	1.1 ± 0.1 ab
		20 μM Fe	16.6 ± 1.0 a	1.4 ± 0.1 a	11.9 ± 0.8 ab	7.0 ± 0.6 a	0.9 ± 0.1 a
	***U. minor***	0 μM Fe	29.1 ± 0.6 b	3.3 ± 0.1 c	12.5 ± 0.3 a	13.0 ± 0.3 bc	2.4 ± 0.0 c
		1 μM Fe	27.4 ± 0.5 b	4.2 ± 0.1 cd	24.0 ± 0.5 c	15.0 ± 0.3 c	4.0 ± 0.1 d
		5 μM Fe	17.2 ± 1.2 a	1.5 ± 0.1 ab	14.7 ± 0.9 ab	7.3 ± 0.6 a	1.7 ± 0.1 c
		20 μM Fe	19.4 ± 1.5 a	2.0 ± 0.1 a	17.7 ± 1.5 cd	7.4 ± 0.3 a	1.5 ± 0.1 bc

**Table 3:** Micronutrient leaf and stem concentrations (mean ± SE; the same letters within the same tissue and element correspond to Tukey’s HSD 95% homogeneous groups).

			[Micronutrients] (mg kg^-1^ DW)				
Tissue	Species	Treatment	Fe	B	Mn	Cu	Zn
**Leaf**	***U. laevis***	0 μM Fe	21.0 ± 0.4 a	96.7 ± 1.9 e	1027.4 ± 20.5 e	17.1 ± 0.3 e	91.3 ± 1.8 d
		1 μM Fe	23.8 ± 5.8 ab	59.1 ± 2.7 d	507.1 ± 13.9 d	10.7 ± 0.9 d	72.2 ± 3.8 bc
		5 μM Fe	37.0 ± 3.6 bc	29.8 ± 1.7 a	256.0 ± 5.5 c	5.4 ± 0.5 bc	48.9 ± 2.1 ab
		20 μM Fe	60.3 ± 2.7 d	30.0 ± 1.8 a	205.5 ± 7.3 b	5.3 ± 0.4 bc	77.6 ± 7.4 cd
	***U. minor***	0 μM Fe	10.8 ± 0.2 a	56.8 ± 1.1 cd	303.8 ± 6.1 c	8.7 ± 0.2 cd	36.5 ± 0.7 ab
		1 μM Fe	11.9 ± 0.1 a	42.4 ± 4.4 bc	278.1 ± 1.2 c	7.0 ± 0.3 c	27.4 ± 2.6 a
		5 μM Fe	38.8 ± 2.3 bc	31.0 ± 2.5 ab	160.8 ± 10.0 a	4.6 ± 0.4 ab	49.7 ± 3.6 ab
		20 μM Fe	44.9 ± 1.5 c	25.8 ± 1.7 a	167.8 ± 11.1 ab	3.5 ± 0.2 a	53.0 ± 3.7 abc
**Stem**	***U. laevis***	0 μM Fe	6.2 ± 0.1 b	13.1 ± 0.3 ab	181.3 ± 3.6 e	9.1 ± 0.2 c	43.9 ± 0.9 a
		1 μM Fe	8.1 ± 0.2 bc	13.0 ± 0.3 ab	61.0 ± 1.2 c	6.1 ± 0.1 bc	26.8 ± 0.5 ab
		5 μM Fe	10.8 ± 1.3 c	10.2 ± 0.8 a	21.9 ± 2.0 a	3.6 ± 0.4 ab	20.2 ± 1.7 a
		20 μM Fe	13.7 ± 1.0 cd	9.8 ± 0.7 a	23.1 ± 2.5 ab	3.2 ± 0.3 a	26.2 ± 2.7 ab
	***U. minor***	0 μM Fe	4.3 ± 0.1 a	14.0 ± 0.3 ab	79.8 ± 1.6 cd	7.0 ± 0.1 c	29.7 ± 0.6 ab
		1 μM Fe	9.0 ± 0.2 c	20.6 ± 0.4 b	88.6 ± 1.8 d	7.7 ± 0.2 c	30.1 ± 0.6 ab
		5 μM Fe	9.9 ± 0.8 c	9.6 ± 0.5 a	31.7 ± 2.2 b	3.7 ± 0.2 ab	27.7 ± 1.7 ab
		20 μM Fe	15.0 ± 0.9 d	10.1 ± 0.6 a	32.2 ± 1.5 b	3.4 ± 0.2 a	32.2 ± 2.5 b

Individual *U. laevis* and *U.minor* seed (extracted from the samara) weighed an average of 6.9 and 5.4 mg DW, respectively. Differences between *U. laevis* and *U. minor* were only recorded for the macronutrients (*g* kg^-1^ DW): N [75.8 versus (vs.) 62.2] K (11.7 vs. 17.3), Ca (1.0 vs. 2.5), and Mg (1.7 vs. 3.0), and the micro-elements (mg kg^-1^ DW): Fe (80.3 vs. 37.7) and Zn (76.9 vs. 56.5). Seeds of both elm species had a Mn concentration around 49.3 mg kg^-1^ DW, which provides evidence for the accumulation of Mn in seedlings during hydroponic culture.

## DISCUSSION

In this study we investigated the root response mechanisms under Fe-limiting conditions of a putative calcicole versus a calcifuge elm species, based on their natural distribution in the soils of the Iberian Peninsula. Calcifuge plants and lichens do not occur in calcareous soils, where their growth and development is largely limited by Fe and P deficiency (Zohlen, 2002; Zohlen and Tyler, 2004; Paul et al., 2009). Calcicole species however, thrive in soils containing a high CaCO_3_ concentration, which implies that they were able to develop efficient mineral element uptake and homeostasis mechanisms as an adaptation to such rhizospheric environment. Since lime-induced chlorosis can sometimes be observed in wild plants grown in calcareous soils and problems of tissue Fe immobilization have been previously reported (Zohlen and Tyler, 1997; Zohlen, 2002), we analyzed for the first time the Fe-reducing and acidification capacity of two *Ulmus* species of major ecological significance in azonal forests of Spain, which are naturally distributed in either siliceous (*U. laevis*) or calcareous (*U. minor*) soils.****

The existing studies on calcifuge and calcicole species were always performed by growing plants in calcareous or acid soils (e.g., Zohlen, 2002; Zohlen and Tyler, 2004) and focused on the distribution and partition of Fe within the different plant tissues (Zohlen and Tyler, 2000). However, plant growth in a solid substrate poses some experimental constraints to analyse in detail the response of roots to Fe-limiting conditions, which led us to carry out the trials in hydroponic culture. While both species have high water requirements as compared to other Mediterranean plants (Galmés et al., 2012; Venturas et al., 2013b), *U. laevis* may be subjected to prolonged soil flooding periods (Collin et al., 2000) in contrast to the occasional flooding that *U. minor* may tolerate (García-Nieto et al., 2000).

The results show that lack of iron (T0) severely affected the growth and development of both elm species. By the end of the experimental period, seedlings grown without Fe were stunted and almost completely defoliated. This shows that Fe is essential and very limiting for *Ulmus *seedling growth and establishment as shown previously for other wild species (Hutchinson, 1967, 1970). Micronutrient contents in leaves and stems of elms grown with no Fe limitation (T20; **Table 3**) were within the range described for other temperate European trees (Hagen-Thorn and Stjernquist, 2005).

Seed nutrient contents may be key for the successful establishment of seedlings (Tyler and Zohlen, 1998), and we observed that *U. laevis* contained twice as much Fe as *U. minor* seeds, in accordance with the higher leaf Chl and Fe concentrations measured in fully expanded leaves. This indicates the higher nutrient requirements of *U. laevis* that may compete better than *U. minor* under flooding, in siliceous soils and with optimal growing conditions. However, for seedling establishment in calcareous soils, it will be important that the root uptake mechanisms of a species are effective for the acquisition of Fe. Our results indicate that under Fe-limiting conditions *U. minor* seems to be more Fe-efficient than *U. laevis*, and hence more competitive. Furthermore, it is likely that fitness and recruitment of calcifuge plants growing in calcareous soils is much reduced due to the occurrence of lime-induced chlorosis. This will yield calcifuge species more susceptible to be displaced by calcicole plants, following a process of natural selection. Thereby, observation of Fe-deficiency symptoms in natural populations is limited to the plants which may be able to withstand a certain degree of Fe-chlorosis when growing in a calcareous, high pH soil at the seedling stage, even though such seedlings may not be competitive.

From the three major root Fe uptake mechanisms characterized in Strategy I plants, we analyzed the root acidification capacity and ferric reductase activity of both elm species in relation to different Fe treatments. Our results suggest that *U. minor* may be better adapted to Fe-limiting environments (i.e., high rhizosphere pH and a lower Fe supply) than *U. laevis*. This can be concluded since the root acidification capacity and reductase activity are higher for *U. minor*, which will subsequently be more efficient in solubilizing and taking up Fe from the rhizosphere. We did not compare the effectiveness of the root ferrous Fe transporter (IRT) between both species, but the increased tissue Mn concentrations determined particularly in *U. laevis* when subjected to Fe-limiting conditions, may provide indirect evidence for the high activity of this transporter and for the interactions between Fe and Mn nutrition (Conte and Walker, 2011; Marschner, 2012).

Iron deficiency changed the nutrient balance of *U. laevis* and *U. minor* seedlings as previously reported for other plant species (e.g., Fernández et al., 2008). We observed that despite growing under Fe-limiting conditions, *U. minor* was able to preserve a better nutrient balance as compared to *U. laevis* seedlings, which experienced more remarkable tissue nutrient variations in relation to Fe deficiency with especial regard to Mn, P, B, and Mg. *U. minor* plants supplied 5 μM Fe reached the largest size, tissue DW and Fe absorption rates, while higher root medium Fe doses improved growth and increased tissue Fe and leaf Chl concentrations in *U. laevis*. This suggests that the calcicole *U. minor* may be adapted to lower soil Fe availability levels as commonly found in calcareous, high pH soils in which the solubility of Fe can be extremely low (Lindsay and Schwab, 1982). In contrast, *U. laevis* appeared to physiologically benefit from receiving a 20 μM Fe concentration via the root system, which may be linked to the generally higher availability of Fe in siliceous soils.

Furthermore, after Fe-resupply, *U. minor* leaves fully re-greened following a homogeneous pattern in contrast to *U. laevis* in which re-greening chiefly occurred on the young leaves. The highest leaf and stem Fe concentration increments after Fe-resupply were also recorded for *U. minor*. Both Chl and tissue Fe results after Fe-resupply may be due to the lower mobility and symplastic uptake of Fe in *U. laevis* as compared to *U. minor*.

The root Fe uptake efficiency of *U. minor* and *U. laevis* may account for their natural distribution in calcareous and acid soils of the Iberian Peninsula, respectively. However, calcifuge species may be affected by the reduced soil availability of other nutrients such as P or Mn (Larcher, 2003). Additional factors such as water availability and drought resistance (Venturas et al., 2013b) may affect the distribution of elms, but our results suggest that the pollen record found in calcareous areas of Eastern Spain probably belongs to the calcicole *U. minor*, since *U. laevis* is largely restricted to western Spain likely due to its calcifuge nature. In contrast, it cannot be *a priori* concluded whether the pollen record in Western Spain (non-carbonated soils) exclusively belongs to *U. laevis*, since calcicole plants may also thrive in siliceous soils.

Our results provide evidence for the important role of Fe as an essential element for growth and survival of a potentially calcifuge species (i.e., *U. laevis*). Phosphorus deficiency of calcifuge plants growing in calcareous soils has however, been suggested to be a major physiological problem (Tyler, 1996; Zohlen and Tyler, 2004). Soil pH and nutrient availability may determine the ecological distribution of plant species (Viani et al., 2014) but further studies correlating soil chemistry, species distribution and physiology shall be performed for clarifying the calcifuge or calcicole nature of *U. laevis* and *U. minor*.

## CONCLUSION

The different response of *U. laevis* and *U. minor* seedlings to Fe-limiting conditions enabled us to interpret their natural distribution in the soils of the Iberian Peninsula. Results concerning the root ferric reductase activity and proton extrusion capacity, together with tissue mineral element concentrations and plant responses to Fe-resupply, provided evidence for the better Fe uptake and homeostasis of *U. minor* as compared to *U. laevis*. The development of hydroponic plant nutrition studies performed with forest species, complemented with field investigations may subsequently prove useful for improving their fitness and establishment during reforestations, and for characterizing nutrient absorption and homeostasis mechanisms of wild plants in relation to their surrounding environment.

## Conflict of Interest Statement

The authors declare that the research was conducted in the absence of any commercial or financial relationships that could be construed as a potential conflict of interest.
